# Supplementary material for: Iterative improvement in the automatic modular design of robot swarms
Source: PeerJ Comput Sci. 2020 Dec 7;6:e322. doi: 10.7717/peerj-cs.322 (PMC7924708; doi:10.7717/peerj-cs.322)
Supplement: Supplemental Information 3 [file peerj-cs-06-322-s003.zip › argos3/doc/api/standalone/a00651_source.html]

ARGoS: plugins/simulator/visualizations/qt-opengl/qtopengl\_lua\_statetree\_model.h Source File


- Main Page
- Related Pages
- Namespaces
- Classes
- Files

- File List
- File Members

# plugins/simulator/visualizations/qt-opengl/qtopengl\_lua\_statetree\_model.h

Go to the documentation of this file.

```
00001 
00007 #ifndef QTOPENGL_LUA_STATETREE_MODEL_H
00008 #define QTOPENGL_LUA_STATETREE_MODEL_H
00009 
00010 namespace argos {
00011    class CQTOpenGLLuaStateTreeModel;
00012    class CQTOpenGLLuaStateTreeVariableModel;
00013    class CQTOpenGLLuaStateTreeFunctionModel;
00014    class CQTOpenGLLuaStateTreeItem;   
00015 }
00016 
00017 extern "C" {
00018 #include <lua.h>
00019 }
00020 
00021 #include <QAbstractItemModel>
00022 #include <QModelIndex>
00023 #include <QVariant>
00024 
00025 namespace argos {
00026 
00027    /****************************************/
00028    /****************************************/
00029 
00030    class CQTOpenGLLuaStateTreeModel : public QAbstractItemModel {
00031 
00032       Q_OBJECT
00033 
00034    public:
00035 
00036       CQTOpenGLLuaStateTreeModel(lua_State* pt_state,
00037                                  bool b_remove_empty_tables,
00038                                  QObject* pc_parent = 0);
00039 
00040       virtual ~CQTOpenGLLuaStateTreeModel();
00041 
00042       virtual QVariant data(const QModelIndex& c_index,
00043                             int n_role) const;
00044 
00045       virtual Qt::ItemFlags flags(const QModelIndex& c_index) const;
00046 
00047       virtual QModelIndex index(int n_row,
00048                                 int n_column,
00049                                 const QModelIndex& c_parent = QModelIndex()) const;
00050       
00051       virtual QModelIndex parent(const QModelIndex& c_index) const;
00052 
00053       virtual int rowCount(const QModelIndex& c_parent = QModelIndex()) const;
00054 
00055       void SetLuaState(lua_State* pt_state);
00056 
00057    public slots:
00058 
00059       void Refresh();
00060       void Refresh(int);
00061 
00062    protected:
00063 
00064       void ProcessLuaState(lua_State* pt_state,
00065                            CQTOpenGLLuaStateTreeItem* pc_item);
00066 
00067       virtual bool IsTypeVisitable(lua_State* pt_state) = 0;
00068 
00069    private:
00070 
00071       lua_State* m_ptState;
00072       CQTOpenGLLuaStateTreeItem* m_pcDataRoot;
00073       bool m_bRemoveEmptyTables;
00074 
00075    };
00076 
00077    /****************************************/
00078    /****************************************/
00079 
00080    class CQTOpenGLLuaStateTreeVariableModel : public CQTOpenGLLuaStateTreeModel {
00081 
00082       Q_OBJECT
00083 
00084    public:
00085 
00086       CQTOpenGLLuaStateTreeVariableModel(lua_State* pt_state,
00087                                          bool b_remove_empty_tables,
00088                                          QObject* pc_parent = 0);
00089 
00090       virtual ~CQTOpenGLLuaStateTreeVariableModel() {}
00091 
00092       virtual QVariant headerData(int n_section,
00093                                   Qt::Orientation e_orientation,
00094                                   int n_role = Qt::DisplayRole) const;
00095 
00096       virtual int columnCount(const QModelIndex& c_parent = QModelIndex()) const;
00097 
00098    protected:
00099 
00100       virtual bool IsTypeVisitable(lua_State* pt_state);
00101 
00102    };
00103 
00104    /****************************************/
00105    /****************************************/
00106 
00107    class CQTOpenGLLuaStateTreeFunctionModel : public CQTOpenGLLuaStateTreeModel {
00108 
00109       Q_OBJECT
00110 
00111    public:
00112 
00113       CQTOpenGLLuaStateTreeFunctionModel(lua_State* pt_state,
00114                                          bool b_remove_empty_tables,
00115                                          QObject* pc_parent = 0);
00116 
00117       virtual ~CQTOpenGLLuaStateTreeFunctionModel() {}
00118 
00119       virtual QVariant headerData(int n_section,
00120                                   Qt::Orientation e_orientation,
00121                                   int n_role = Qt::DisplayRole) const;
00122 
00123       virtual int columnCount(const QModelIndex& c_parent = QModelIndex()) const;
00124 
00125    protected:
00126 
00127       virtual bool IsTypeVisitable(lua_State* pt_state);
00128 
00129    };
00130 
00131    /****************************************/
00132    /****************************************/
00133 
00134 }
00135 
00136 #endif
```

---

Generated on 10 Jul 2018 for ARGoS by 
 1.6.1
